# Feasibility of robotic-assisted pancreatic resection in patients with previous minor abdominal surgeries: a single-center experience of the first three years

**DOI:** 10.1186/s12893-022-01525-y

**Published:** 2022-03-04

**Authors:** Paul Viktor Ritschl, Hannah Kristin Miller, Karl Hillebrandt, Lea Timmermann, Matthäus Felsenstein, Christian Benzing, Brigitta Globke, Robert Öllinger, Wenzel Schöning, Moritz Schmelzle, Johann Pratschke, Thomas Malinka

**Affiliations:** 1 Department of Surgery, Campus Charité-Mitte and Campus Virchow-Klinikum, Charité – Universitätsmedizin Berlin, corporate member of Freie Universität Berlin, Humboldt-Universität zu Berlin, and Berlin Institute of Health, Berlin, Germany; 2 Clinician Scientist Program, Berlin Institute of Health (BIH), Anna-Louisa-Karsch-Str. 2, 10178 Berlin, Germany; 3 Humboldt-Universität Zu Berlin, Berlin, Germany; 4 Berlin Institute of Health, Berlin, Germany; 5 Clinician Scientist Program, Berlin Institute of Health (BIH), Anna-Louisa-Karsch-Str. 2, 10178 Berlin, Germany

**Keywords:** Robotic-assisted pancreatic surgery, daVinci Xi system, History of abdominal surgery

## Abstract

**Background:**

Robotic-assisted pancreatic surgery is limited to specialized high-volume centers and selected patient cohorts. Especially for patients with a history of previous abdominal surgeries, the standard procedure remains open surgery due to the fear of complications caused by abdominal adhesions.

**Methods:**

Clinical data of all consecutive patients undergoing robotic-assisted pancreatic surgery using the daVinci Xi system (Intuitive Surgical) at our center (Department of Surgery, Universitätsmedizin Berlin, Germany) were collected prospectively and further analyzed from October 2017 to October 2020. Prior abdominal surgeries were specified according to the surgical approach and localization. In univariate and multivariate analysis, baseline and perioperative parameters of patients with a history of prior abdominal surgeries (PS) were compared to those of patients with no history of prior abdominal surgeries (NPS).

**Results:**

Out of 131 patients undergoing robotic-assisted pancreatic surgery, 62 (47%) had a history of abdominal surgery. Previous procedures included most often appendectomy (32%) followed by gynecological surgery (29%) and cholecystectomy (27%). 24% of PS had received multiple surgeries prior to the robotic-assisted pancreatic resections. Baseline characteristics and comorbidities were comparable between the groups. We did not detect differences in the duration of surgery (262 min), conversion rates (10%), and postoperative complications between NPS and PS. Postoperative pancreatic fistula (POPF), postpancreatectomy hemorrhage (PPH), and in-house mortality showed no significant differences between the two groups. Multivariate analysis revealed male sex and high BMI as a potential predictive factor for severe postoperative complications. Other characteristics like the type of pancreatic resection, ASA, and underlying malignancy showed no difference in the multivariable analysis.

**Conclusions:**

We propose robotic-assisted pancreatic surgery to be safe and feasible for patients with a history of minor prior abdominal surgery. Hence, each patient should individually be evaluated for a minimally invasive approach regardless of a history of previous operations.

## Introduction

Today, pancreatic surgery is still associated with a high risk of postoperative complications despite substantial improvements in surgical techniques and the performance of surgery in high-volume centers [1–3]. Although no generally accepted definition of high-volume centers exists (range > 10 to > 100), it is well accepted that with increasing annual numbers of major pancreas resections the quality improves [4, 5]. At present, notwithstanding developments in pharmacologic and oncologic treatment, surgical resection is still the primary treatment of benign and malignant pancreatic lesions.

In 1909 Walter Kausch established the first steps in pancreaticoduodenectomy. After Kausch died in 1935, Whipple took the first procedure and defined the pancreaticoduodenectomy, called “Kausch-Whipple Operation” and thereby founded modern pancreatic surgery [6, 7]. However, due to high rates of complication, pancreaticoduodenectomy was performed infrequently in the middle of the last century [6]. During the following decades, high volume centers developed, and the mortality rate decreased to five percent [8, 9]. Despite substantial improvements, the rate of postoperative complications, such as postoperative pancreatic fistula (POPF), postoperative pancreatic hemorrhage (PPH), surgical site infections (SSI), insufficiency of the implemented anastomoses, and pulmonary complications, remains a significant issue [1–3].

In the last decade, minimally invasive techniques have revolutionized modern surgery in general by reducing postoperative morbidity and mortality [10]. Several studies demonstrated the known benefits of minimally invasive surgery with comparable rates of perioperative mortality and morbidity [5, 11, 12]. The benefits of a minimally invasive approach are based on reduced trauma to the abdominal wall reducing blood loss, postoperative pain, and surgical site infections (SSI), leading to better and faster postoperative recovery [10, 13, 14]. Consecutively, in the early 90 s, the first laparoscopic partial pancreaticoduodenectomy (LPD) was performed [15]. In the following, laparoscopy has also been introduced to the partial pancreaticoduodenectomy. Due to several limitations, laparoscopy is currently mostly limited to distal resections [10, 16]. After its implementation, robotic assistance compensated for several disadvantages of laparoscopy: It provides a three-dimensional view and more sophisticated instrument manipulations, reduces tremor transmission, and allows for up to seven degrees of freedom. Therefore, more complex oncological operations became safe and feasible [17, 18]. A recent meta-analysis comparing robotic-assisted resection with laparoscopic resection showed that the use of robotic assistance leads to fewer blood transfusions, lower conversion rates, shorter procedure times, and lower total costs, thereby confirming its superiority in complex oncologic surgery [19]. Nevertheless, adhesions after previous abdominal surgeries may hinder the success and practicability of robotic-assisted pancreatic resection. Abdominal adhesions are well known to prolong surgery time, and adhesiolysis is associated with iatrogenic bowel injury [20]. The goal of the following analysis was to investigate the impact of prior abdominal surgeries on perioperative complications and conversion rates in robotic-assisted pancreatic surgery.

## Methods

### Data collection

A prospective analysis of all consecutive cases of robotic-assisted pancreatic surgery between October 20th of 2017 and October 20th of 2020 was performed at the Department of Surgery, Charité—Universitätsmedizin Berlin. All patients were included in a post-marketing study (DRKS00017229). All resections were carried out using the daVinci Xi surgical system© (Intuitive, Sunnyvale, CA, USA). The presented study was performed according to the Declaration of Helsinki and approved by the independent institutional review board of the Charité-Universitätmedizin Berlin (EA4/084/17). All participants have provided written consent.

Patients that underwent previous abdominal surgery (PS) were compared to patients without previous abdominal surgery (NPS). We categorized the procedures into upper and lower abdominal surgeries to consider the most likely location of intra-abdominal adhesions. Furthermore, cases of open and laparoscopic surgeries and multiple surgeries were distinguished.

The primary objective of the present study were perioperative complications and mortality. Complications were classified according to the Clavien/Dindo-classification [21].

Postoperative pancreatic fistula (POPF), postoperative hemorrhage, and delayed gastric emptying (DGE) were defined according to ISGPS definitions [22–25]. General patient characteristics such as age, sex, underlying pathology (malignant or benign nature of pancreas lesions), and overall physical status using the American Society of Anesthesiologists’ Physical Status Classification (ASA score) were considered to determine preoperative differences between the two groups. In addition, the type of pancreatic resection was analyzed, distinguishing between enucleation of the tumor, left resection with and without splenectomy, Appleby procedure, PPPD, and total pancreatectomy to determine differences in each subgroup. Besides complications, conversion rates from laparoscopic to open surgery, duration of surgery, intensive care unit (ICU) length of stay, and hospital length of stay (LOS) were considered relevant endpoints.

### Surgical technique and perioperative management

In the case of underlying malignancy, each case was individually discussed in our multidisciplinary tumor conference. The surgical team predefined the surgical approach (open, laparoscopic, or robot-assisted) in agreement with the patient. As this prospective study was not primarily evaluating the surgical approach, there were no standardized selection criteria for open, laparoscopic, or robot-assisted surgeries. Furthermore, our standard procedure contains preoperative computed tomography or magnetic resonance imaging, including chest, abdomen, and pelvis imaging for preoperative staging, including an angiography of the vessels. The same surgical team consisting of two experienced pancreatic surgeons performed all surgeries using the daVinci Xi surgical system (Intuitive, Sunnyvale, CA, USA). Our standard operating procedure for robot-assisted pancreas resection has just been published elsewhere [18, 26]. After surgery, most patients were routinely observed in ICU.

### Statistical method

Patient characteristics were examined with descriptive statistics (using frequencies and percentages). To compare categorical variables between NPS and PS groups, we used the Pearson chi-square test for categorical data, the t-test for continuous parametric data (displayed by mean and standard deviation), and the Mann–Whitney U test for nonparametric continuous data (displayed by median and interquartile range). Multivariate regression analysis was performed using a binary logistic regression model for categorical dependent variables.

A p-value < 0.05 was considered statistically significant. IBM SPSS Statistics was used for all statistics.

## Results

### Study cohort and previous abdominal surgeries

The analysis included 131 robotic-assisted pancreatic resections between October 2017 and October 2020. The study cohort was split into 69 patients (53%) with no prior surgery (NPS) and 62 (47%) with prior surgery (PS). All types of pancreatic resections (enucleation, left resection with or without splenectomy, Appleby procedure, total pancreatectomy, and PPPD procedure) have been included. Operations were all performed at the Department of Surgery, Campus Virchow-Klinikum, Charité—Universitätsmedizin Berlin.

To analyze the influence of the type of prior surgeries on the outcomes of robotic pancreatic resections, we divided the patients with prior surgery into subgroups (location, open versus laparoscopic surgery). Forty-eight patients have had operations in the lower abdomen (77% of PS), mostly appendectomies (n = 20; 32%) or gynecological surgeries (n = 18; 29%), 19 in the upper abdomen (31%), with 17 of them operated by cholecystectomy (Table 1). In 15 (24%) cases, patients had undergone more than one previous abdominal surgery and were therefore accounted in more than one group. Regarding the surgical technique of prior surgeries, 29 (47%) were reported as minimally invasive (laparoscopic), 34 (55%) as open surgeries.

**Table 1:** List of previous surgeries

Type of prior surgery	n	% of PS
Any abdominal surgery	62	100
Upper abdomen	19	31
CCE	17	27
Fundoplication	1	2
Left Nephrectomy	1	2
Lower abdomen	48	77
Appendectomy	20	32
Gynecological surgeries	18	29
Hernias	13	21
Prostatectomy	2	3
Rectumresection	1	2
Aortofemoral Bypass	1	2
Laparoscopic surgery	29	47
Open surgery	34	55
Multiple surgeries	15	24

Patients may have received multiple surgeries in the past and were therefore assigned to more than one group. *PS*  previous surgery

### Patient characteristics

Overall, both subgroups were comparable regarding characteristics like sex, age, BMI, and physical status (ASA score) (Table 2). The indication for surgery was similar within the groups: in the NPS group, the percentage of malignant tumors was 62% compared with 57% in the PS group (p = 0.495). Analyzing the comorbidities in both groups showed that diabetes and cardiovascular disease were the most common comorbidities with no difference between the two groups.

**Table 2:** Characteristics and Indication for surgery

	NPS (n = 69)	PS (n = 62)	p-value
Sex (female)	29 (42%)	34 (55%)	0.143
Age	56 ± 13	66 ± 12	0.436
BMI	25 ± 4	26 ± 5	0.223
ASA score ≥ 3	21 (30%)	23 (37%)	0.133
Malignant diagnosis	43 (62%)	35 (57%)	0.495
Comorbidities	57 (83%)	58 (93%)	0.056
Cardiovascular	36 (52%)	39 (63%)	0.215
Diabetes	12 (17%)	7 (11%)	0.322
Pulmonary	6 (9%)	11 (18%)	0.124
Renal insufficiency	1 (1%)	2 (3%)	0.497
Other	52 (75.4%)	51 (82%)	0.336
OP specifics			
Type of pancreas resection			0.022
Enucleation	2 (3%)	0 (0%)	
Spleen-preserving distal pancreatectomy	3 (4%)	1 (2%)	
Distal pancreatectomy with splenectomy	20 (29%)	33 (53%)	
Appleby procedure	3 (4%)	1 (2%)	
Total pancreatectomy	5 (7%)	0 (0%)	
PPPD/Whipple procedure	36 (52%)	27 (44%)	

*NPS* no previous surgery, *PS* previous surgery. ASA score = American Society of Anesthesiologists’ Physical Status Classification; patients may suffer from more than one comorbidity

The only significant difference between the groups was found by comparing the type of pancreatic resections (Table 2). Left resection with splenectomy was done in 33 (53%) in the PS group versus 20 (29%) procedures in the NPS group. PPPD/Whipple procedure was done in 36 (52%) in the NPS group versus 27 (44%) in the PS group.

### Perioperative outcomes

In general, no significant differences were found when analyzing the most relevant outcome parameters of the investigated study cohorts (Table 3). Robotic-assisted pancreatic resection in the NPS group lasted in a median of 262 min (95% CI 167–331) compared to 245 min (95% CI 150 – 301) in the PS group (p = 0.167).

**Table 3:** Perioperative outcome in patients undergoing robotic assisted pancreatectomy with or without previous abdominal surgeries

	NPS (n = 69)	PS (n = 62)	p-value
Operating time (min)	262 (167–331)	245 (150–301)	0.167
Conversion	7 (10%)	4 (7%)	0.447
Complications: Clavien–Dindo	48 (70%)	40 (65%)	0.900
1	4 (6%)	3 (5%)	
2	3 (4%)	6 (10%)	
3a	8 (12%)	6 (10%)	
3b	17 (24%)	15 (24%)	
4a	8 (12%)	6 (10%)	
4b	2 (3%)	1 (1%)	
5	6 (9%)	3 (5%)	
POPF yes/no	30 (43%)	23 (37%)	0.457
Biochemical leakage	6 (9%)	6 (10%)	
B	21 (30%)	16 (26%)	
C	3 (4%)	1 (2%)	
PPH	10 (14%)	9 (15%)	0.201
A	3 (4%)	1 (2%)	
B	6 (9%)	2 (5%)	
C	1 (1%)	5 (8%)	
SSI	26 (37%)	23 (37%)	0.293
Superficial	1 (1%)	4 (6%)	
Intraabdominal fluid collection	25 (36%)	19 (31%)	
DGE	7 (10%)	6 (10%)	0.934
Insufficient Choledochojejunostomy	5/41 (12%)	1/27 (4%)	0.176
Pulmonary complication	14 (20%)	12 (19%)	0.893
ReOP	8 (12%)	6 (10%)	0.684
90 days mortality	6 (9%)	3 (5%)	0.384
Length of ICU stay (days)	1 (1–3)	1 (1–2)	0.950
Length of hospital stay (days)	13 (9–24)	11 (8–17)	0.185
Hospital readmission	12 (17%)	11 (18%)	0.958

*ASA score*  American Society of Anesthesiologists’ Physical Status Classification, *DGE*  delayed gastric emptying, *PG*  pancreaticogastrostomy, *PPH*  postpancreatectomy haemorrhage, *PS*  previous surgery, *SSI*  surgical side infection, patients may have had more than one complication

There were eleven cases of conversion to open or conventional laparoscopic surgery in our cohort, seven (10%) in the NPS group and four (7%) in the PS group (p = 0.447). Complications, classified by the Clavien–Dindo classification, showed no significant difference between the NPS and the PS group (p = 0.900). 67% of all patients (n = 88/131) have had complications, including mild complications (Clavien–Dindo 1–3a) and severe complications (Clavien–Dindo > 3a). In particular, main complications like postpancreatectomy hemorrhage (p = 0.201), POPF (p = 0.457), insufficient PG (p = 0.612) or insufficient BDA (p = 0.176), pulmonary complications (p = 0.893), surgical site infections (p = 0.293), delayed gastric emptying (p = 0.934), and the necessity of reoperation (p = 0.684) were found to be similarly distributed among patients with or without a history of previous abdominal surgeries. Overall, 90 days mortality after robotic-assisted pancreas resection was 6.8% (n = 9/131). The rates were comparable in both study groups (p = 0.384). Furthermore, in-hospital as well as ICU length of stay was comparable between the investigated groups (ICU: PS 1d (95% CI 1–2) vs NPS 1d (95% CI 1–3), p = 0,950; hospital: PS 11d (95% CI 8–17) vs NPS 13d (95% CI 9–24), p = 0.185). Similarly, hospital readmission also occurred at a comparable rate (p = 0.958). The outcome data are summarized in Table 3.

In an additional analysis, different subtypes of prior abdominal surgeries were compared to the NPS group (Table 4). Neither location (lower/upper abdomen), surgical approach (laparotomy/laparoscopy), nor a history of multiple previous surgeries showed significant differences in outcome parameters like surgery time, complication rate, length of hospital stay, or conversion rate. Hence no high or low-risk groups within PS could be identified.

**Table 4:** Perioperative outcome in patients undergoing robot-assisted pancreas resection with subgroup analysis of different previous surgeries

	NPS (n = 69)	uaPS (n = 19)	p-value*	laPS (n = 48)	p-value*	openPS (n = 34)	P-value*	multiPS (n = 15)	p-value*
Operating time (min)	262 (167–331)	244 (162–310)	0.742	246 (149–300)	0.110	226 (148–274)	0.080	258 (179–299)	0.404
Conversion	7 (10%)	0 (0%)	0.148	4 (8%)	0.741	4 (12%)	0.802	1 (7%)	0.677
Complications: Clavien–Dindo									
> 3a	33 (48%)	10 (53%)	0.711	19 (40%)	0.377	11 (32%)	0.135	8 (53%)	0.699
POPF yes/no	30 (43%)	8 (42%)	0.742	17 (35%)	0.704	11 (32%)	0.527	6 (40%)	0.798
Biochemical leakage	6 (9%)	1 (5%)		5 (10%)		4 (12%)		2 (13%)	
B	21 (30%)	7 (37%)		11 (23%)		6 (18%)		4 (27%)	
C	3 (4%)	0 (0%)		1 (2%)		1 (3%)		0 (0%)	
Postpancreatectomy hemorrhage	10 (14%)	4 (21%)	0.774	7 (15%)	0.220	3 (9%)	0.240	2 (13%)	0.936
A	3 (4%)	1 (5%)		1 (2%)		0 (0%)		1 (7%)	
B	6 (9%)	2 (11%)		2 (4%)		1 (3%)		1 (7%)	
C	1 (1%)	1 (5%)		4 (8%)		2 (6%)		0 (0%)	
Intraabdominal fluid collection	25 (36%)	9 (47%)	0.567	14 (29%)	0.301	6 (18%)	0.36	5 (33%)	0.766
Pulmonary complication	14 (20%)	4 (21%)	0.942	8 (17%)	0.622	3 (9%)	0.140	2 (13%)	0.534
ReOP	8 (12%)	0 (0%)	0.105	6 (13%)	0.898	2 (6%)	0.368	1 (7%)	0.570
90 days mortality	6 (9%)	1 (5%)	0.624	2 (4%)	0.340	2 (6%)	0.616	0 (0%)	0.236
Length of ICU stay (days)	1 (1–3)	1 (1–4)	0.964	1 (1–2)	0.814	1 (1–2)	0.592	1 (1–8)	0.587
Length of hospital stay (days)	13 (9–24)	11 (8–16)	0.288	10 (8–18)	0.230	10 (8–14)	0.61	10 (8–32)	0.652

*NPS* no previous surgery, *uaPS* upper abdominal previous surgery, *laPS*  lower abdominal previous surgery, *openPS*  open previous surgery, *multiPS*  multiple abdominal previous surgeries; individuals may be part of more than one subgroup

*Compared to NPS

### Multivariate analysis

Furthermore, a logistic regression analysis was performed concerning severe postoperative complications (Clavien–Dindo > 3a; see Table 5). The multivariate binary logistic regression model showed that higher BMI is significantly associated with severe postoperative complications. On the other hand, the female sex was shown to be protective against severe complications (OR 0.410, p = 0.038). Procedure time, the type of pancreatic resection, and history of previous abdominal surgery demonstrated not to affect the regression model.

**Table 5:** Multivariate binary logistic regression analysis for potential predictive factors for severe postoperative complications (Clavien–Dindo > 3a)

	p-value	Odds ratio	95% CI for Exp(B)
Previous abdominal surgery	0.237	–	0.233–1.434
Operating time (min)	0.119	–	0.988–1.001
Conversion	0.137	–	0.688–15.112
Age	0.269	–	0.985–1.057
Sex (female)	0.038	0.410	0.177–0.950
BMI	0.001	1.230	1.092–1.385
ASA	All n.s	–	
Malignant diagnosis	0.112	–	0.217–1.174
Comorbidity	0.154	–	0.103–1.432
Type of pancreas resection	All n.s	–	

*ASA score*  American _Society of Anesthesiologists’ Physical Status Classification; *n.s.* = not significant, patients may suffer from more than one comorbidity

## Discussion

Prior abdominal surgery is a commonly mentioned factor to prefer open procedure rather than the laparoscopic approach in the past. We demonstrate that robot-assisted pancreas resection can be performed safely even after previous abdominal surgery. Neither intraoperative required conversion nor time of surgery was affected by a positive history of abdominal surgery. To the best of our knowledge, there has been no other systematic study focusing on the correlation of prior abdominal surgery and the robot-assisted approach to pancreatic surgery.

Over the past three decades, minimally invasive surgical procedures have gained wide acceptance among patients and surgeons because of their safety and improved surgical outcomes. From the starting point of the first laparoscopic surgeries as early as 1910, it took almost a century and countless developing steps (technically and medically) to the latest robotic-assisted Whipple procedure [27]. This revolution is carried by numerous published studies demonstrating the superiority of the laparoscopic approach in general, emphasizing less blood loss and rapid recovery [28–31]. On the other side, no randomized controlled trial (RCT) has yet been published on robot-assisted surgery versus open surgery in the field of pancreas resection [32].

However, in the decision-making process of open versus minimal invasive resection, previous abdominal procedures are often seen as contraindications due to suspected abdominal adhesions. However, our study showed no significant differences between the groups regarding severity of complications, intensive care unit or in-hospital stay, or any other relevant outcome parameter. Subgroup analysis did not reveal any specific group of previous surgeries (open/multiple/upper abdomen) with a higher risk for complications. It must be mentioned that within this group, the extent of previous surgeries was limited to appendectomies, cholecystectomies, or gynecological surgeries. For this reason, this current study may not directly be relatable to recent reports of increased time of surgery after previous major liver resections. A recently published study of liver resections showed that previous liver surgery, which may be considered major surgery, was associated with a significantly longer time of surgery but still had a similar complication rate [17].

Looking at specific complications related to pancreatic resection, we could demonstrate that DGE, POPF, and PPH were comparable in patients with or without previous abdominal surgeries. This is in line with recent studies on open versus laparoscopic surgery, which have shown that laparoscopic pancreaticoduodenectomy has identical risk and, in some cases, a lower risk of developing POPF than open surgery [33, 34].

However, according to recently published multinational registries, the minimally invasive pancreas resection rate is still far below 15% [35]. Hence, some hurdles prevent its wide application. On the one hand, the reason for that may be that pancreatic surgery still ranks among the most complex abdominal procedures with high morbidity and mortality [16]. Therefore, more than in other surgical fields, it is necessary to have centers of excellence with sufficient amounts of resections per year. Just with an adequate caseload, the development of minimal invasive skills and training of fellows becomes possible [5, 36, 37].

Another reason for the low numbers of minimally invasive procedures in pancreatic resection is undoubtedly due to a selection bias. However, as known from other laparoscopic procedures, it is especially the old, multimorbid patients that profit the most from reduced trauma to the abdominal wall [38]. Patients with prior abdominal surgeries might be perceived as too complex for similar reasons. Despite a history of previous abdominal surgery, the applicability of a pneumoperitoneum during the performed surgery, e.g., due to cardiovascular comorbidities, might serve as an explanation.

Our study has several limitations. First, although the presented study is a prospective observational study, it has an inherent selection bias. As robotic-assisted surgeries and not consecutive cases define the cohort, open pancreas resections are missing. Accordingly, it is not reasonably possible to assess the reasons for or against a minimally invasive approach per patient retrospectively. Secondly, due to cohort size, the power of subgroup analysis might be too low. Hence, specific surgical histories, especially major surgeries (e.g., liver transplantation and gastrectomy), have not been in our cohort and, of course, might affect the postoperative outcome after robotic-assisted pancreas resection.

In conclusion, our study indicates that a history of minor abdominal surgery is not associated with longer operative time or postoperative complications, including pancreatic fistula or hemorrhage in robotic pancreatic surgery. Therefore, we suggest that robotic-assisted surgery should be considered in all patients undergoing pancreatic surgery regardless of previous surgery. Furthermore, in upcoming RCTs about the relevance of robotic assistance in pancreatic surgery, special attention should be paid to patients with a history of previous abdominal surgeries.

## Data Availability

The datasets used and analyzed during the current study are available from the corresponding author on reasonable request.

## Abbreviations

ASA: American Society of Anesthesiologists
ICU: Intensive care unit
LPD: Laparoscopic partial pancreaticoduodenectomy
LOS: Length of hospital stay
NPS: No prior surgery
POPF: Postoperative pancreatic fistula
PPH: Postpancreatectomy haemorrhage
PS: Prior surgery
SSI: Surgical site infection
